# Annotations

**Published:** 1892-06-25

**Authors:** 


### ANNOTATIONS.

**After Thoughts.**

THE amounts collected by the various churches and chapels on behalf of the Hospital Sunday Fund, are beginning to be sent in, and it strikes us that there is an unusual number of second and later subscriptions for large amounts sent in by private individuals. Out of a total of £5,000, reported on Tuesday morning, no less a sum than £550 appears as second subscriptions by private persons. It would seem possible that those donors may have thought the subject out at home, after listening to the Sunday sermon in church, and their second thoughts appear to have been of the most generous character. It will be several weeks before all the Sunday collections are sent in, and the amounts published. There is, therefore, plenty of time for other people, as well as those named, to give the subject a little additional consideration. *The Speaker*, in an excellent article, on Saturday last, stated that we hear a great deal of "hospital scandals," but, in its judgment, the greatest scandal was the impecuniosity into which the public have allowed the metropolitan hospitals to fall. Lord Sandhurst, the Chairman of the Royal Commission on Hospitals, stated at the annual meeting of the Hospitals Association last Saturday that the Commission had found the voluntary hospitals of London to be "very satisfactory"; and that nowhere in the world were hospitals of such excellence to be found unless it was in the case of one single institution at Berlin. These are excellent reasons for "After Thoughts," and we hope they may induce many readers to double, treble, and quadruple their previous subscriptions before the final accounts of the Sunday Fund are made up.

**Home Reading.**

"READING maketh a full man," Bacon tells us. It is very necessary to be a "full man" in these competitive times; and if even it were not necessary from a competitive point of view, there is a great deal of pleasure in only making the attempt. The man who is not a reader hardly knows he is born. The English as a nation have never been wide readers; but those among them who have read, have read with conspicuous intelligence. They have been masters of their reading, not its slaves. The best thinkers see before this age, and in the near future, a very different state of things from that which has prevailed for many centuries. The people are taking the government of their states into their own hands. We have it on high authority that "War is a game which, if peoples were wise, kings would not play at." Peoples are now having the chance of showing whether they are "wise" or not. It is assumed by many that they will be wise in the near future, and that the coarse, vulgar, and unscrupulous ways of living which are encouraged by warfare will soon cease. On the other hand, since man is a fighting animal, other kinds of conflict will take the place of the methods of the cannon and the sword. Intellectual conflict is to be the chief warfare of the future. The men of the most effective intellect will be the leaders and the commanders. Those of poor intellect, or whose intellect is poorly cultivated, will constitute the rank and file, or even the camp followers. The National Home Reading Union, in anticipation of the wants of the ordinary Englishman, is setting before its constituents and the country a scheme of reading and culture which will make every reader of moderate industry a man of intelligence, and even of some real culture. We have not space to publish details of the lectures that are now being given, or of the systematic courses of reading that are suggested by the Union, and that are carried out by local societies in many of the large and small towns of the kingdom. But we emphatically commend the admirable work which the association is doing, and express the firm conviction that if every large and small centre of population in the kingdom had an efficiently-worked branch of the Home Reading Union in its midst, a peaceful revolution of the most civilised and advantageous character would be gradually wrought in our whole population. The London centre of the society is at Surrey House, Victoria Embankment, W.C.

**The Health of Halifax.**

VERY few people are aware of the amount of thought and labour expended on the health of the people by the public authorities in these times of advancing civilisation. It is probable that nine-tenths of the people of Halifax who have been favoured with the annual report of Dr. Ainley, the Medical Officer of Health for the borough, have put it into the waste-paper basket as a document much too technical and tedious to be read. And yet to those who know how to read such a report it is not only as interesting as a novel, but is really a novel. It gives the story of the struggles and tragedies of a thickly-peopled borough during a single year. A chart, for example, shows at a glance how the angel of death hovered over the town from January to December, and, with the plagues of tubercle, influenza, and common disease in his hand, smote his scores in January, his hundreds in May and June, and his dozens in September and October. The story of the deaths of infants is truly pathetic. When we read that a hundred and seventy-two per thousand of those under one year of age died, we get a glimpse of poverty, unwholesomeness, miserable homes, ignorant minds, and suffering hearts which would appal us if we should dwell upon it too long. Dr. Ainley strives, and very successfully, to instruct the thoughtful people in his borough on the two fundamentally important subjects of the causes of death and the prevention of disease. He gives them in a few words the reasoned conclusions of the eminent specialists, who discussed the question of tuberculosis at the recent International Congress of Health. He tells them that human and bovine tuberculosis are identical, that the drinking of tuberculous milk and the eating of tuberculous meat without the most thorough cooking of both is dangerous, and he reminds them of the striking fact that of all the carcases of beef examined by sanitary inspectors, one in every six is found to be tuberculous. The amount of detailed work carried out by Dr. Ainley, and by Mr. David Travers (the chief sanitary inspector), and Mr. John Hutchinson (the meat inspector), with their several assistants, is really striking, and cannot but be of the greatest service to the well-being of the borough. Every part of the town and district is systematically visited during the year, as many as 25,633 visits being paid by the several inspectors. It is hardly possible to believe that more than three thousand defective drains in private houses should require attention in a town like Halifax, with a population of a little over eighty thousand, in a single year; and equally difficult of credit is it that over thirteen thousand nuisances should require to be reported. But hundreds of details go to the making up of these figures, showing, as we have said, an amount and thoroughness of work of which the unheeding public knows little or nothing. Space forbids us to do more at present than to congratulate Dr. Ainley on a most full and intelligent report, and to suggest that every thoughtful person in Halifax should make a point of studying it.

